# Protective Effect of Ginger oil on Aspirin and Pylorus Ligation-Induced Gastric Ulcer model in Rats

**DOI:** 10.4103/0250-474X.58195

**Published:** 2009

**Authors:** M. Khushtar, V. Kumar, K. Javed, Uma Bhandari

**Affiliations:** Department of Pharmacology, Faculty of Pharmacy, Hamdard University, New Delhi-110062, India; 1 Department of Chemistry, Faculty of Science, Hamdard University, New Delhi-110062, India

**Keywords:** Gastric ulcer, ginger oil, γ-GTP, aspirin and pyloric ligation

## Abstract

The present investigation was performed in aspirin and pylorus ligation-induced ulcer model in Wistar rats, in which ability of ginger oil to provide gastric protection was studied at two different doses, 0.5 and 1 g/kg po. Gastric protection was evaluated by measuring the ulcer index, serum γ-GTP levels, total acidity of gastric juice and gastric wall mucus thickness. The results obtained in the present study indicated that ginger oil has a protective action against gastric ulcers induced by aspirin plus pylorus ligation in Wistar rats.

Peptic ulcer is worldwide problem and its prevalence is quite high in India. Several field studies from different parts of our country suggest its occurrence in 4 to 10 per thousand populations. Three states of India viz. Tamil Nadu, Andhra Pradesh, and Jammu and Kashmir are considered to be very high risk areas. The exact cause of peptic ulcer is not known, the disease results in chronic suffering, loss of working hours and occasional fatality. Smoking, alcoholism, and spices add to the severity of the disease that often precipitate serious complication of ulcer[1].

Over the past few decades, there has been surge in research activity aimed towards the development of effective and safe antiulcer drugs both synthetically and from natural resources. Reports on clinical evaluation of synthetic drugs show that there are incidences of relapses and danger of drug interactions during ulcer therapy. Hence, the search for new and ideal antiulcer drug continues and has also been extended to herbal in search for new and novel molecules, which afford better protection and decrease the incidence of relapse. Further, herbal drugs mostly augment the defensive factors such as mucin secretion, cellular mucus, bicarbonate secretion, mucosal blood flow and cell turnover[2].

Ginger (*Zingiber officinale* Roscoe) is one of the most commonly used herbal supplements and its substantial use in folk remedies for different medical conditions has been documented. Traditionally, ginger has been used to treat a wide range of ailments including gastrointestinal disorders, such as stomachaches, abdominal spasm, nausea, and vomiting, as well as in arthritis and motion sickness[3,4].

The dried rhizome of ginger contains approximately 1-4% volatile oils. These are the medicinally active constituents of ginger, and they are also responsible for ginger's characteristic odour and taste[5]. Phytochemical studies showed that the plant is rich in a large number of substances, including zingiberene, bisabolene, gingerols and shogaols[6,7]. These compounds have been reported to display diverse biological activities such as antioxidant[6], antiinflammatory[8–10]] and anticarcinogenic properties[11]. They also exhibited a spasmolytic activity, which is mediated via blocking Ca^2+^ channels[12]. A number of recent studies have renewed interest in ginger for the treatment of chronic inflammatory conditions[13,14]. Al-Yahya *et al,*[15] has reported the gastroprotective activity of ginger in Wistar rats.

Significant new information has been forthcoming on the pathogenesis of various types of drug-induced ulcers and great strides in our basic understanding of gastro-duodenal physiology and hormonal control have been undertaken. However, mechanism underlying the gastroprotection has not been well understood[16]. The brush border enzymes, gamma glutamyl transpeptidase (γ-GTP) is involved in the transfer of amino acids across the cellular membrane and in glutahione metabolism. γ-GTP is found in high concentrations in the liver, bile ducts and kidney. The focus has been on the role of γ-GTP in the mucosal defense aspect in the etiology of peptic ulcer disease[17].

As no reports are available on the possible antiulcer effects of the ginger oil, the present work was carried out to study the effect of ginger oil in experimentally-induced gastric ulcers in rats and their possible mechanisms of action by studying their effects on various mucosal offensive and defensive factors.

Aspirin was purchased from GS Chemicals, Mumbai, India. Omeprazole was procured from Ranbaxy Laboratories, New Delhi, India. Ginger oil was obtained as a gift sample from Som Extracts, Ghaziabad, India. All other chemicals used were of analytical grade.

The experimental protocol was approved by the Institutional Animal Ethics Committee (IAEC) of Hamdard University, New Delhi. Adult Wistar rats of either sex, weighing 150-200 g, were procured from the Central Animal House Facility, Hamdard University, New Delhi. The animals were kept in polypropylene cages (8 in each cage) under standard laboratory conditions (12 h light and 12 h dark cycle) and had free access to commercial pellet diet (Amrut rat feed, Nav Maharastra Chakan Oil Mills Ltd, Delhi, India) and tap water *ad libitum*.

Aspirin was suspended in 1% CMC solution and administered orally in the dose of 200 mg/kg in non fasted rats once daily for 5 days. Ginger oil and omeprazole were administered orally to the respective treatment groups 30 min before each aspirin treatment where as the control group received only vehicle (1% CMC solution). On the 6th day, pylorus ligation[18] was performed under ether anaesthesia on 36 h fasted rats, immediately after pylorus ligation aspirin treatment was given. Drinking water was withheld after pylorus ligation on 6^th^ day in each rat and gastric juice was allowed to accumulate for a period of 4 h. After that blood samples (2-3 ml) were collected from the retro-orbital plexus of all groups using microcapillary tube.

About 2-3 ml of blood was collected in a sterile serum vacutainer and kept undisturbed at 37° for 45 min. During this period, serum exuded and the clot retracted. The serum was aspirated using a sterile pipette after centrifugation at 3000 rpm for 15 min. The collected serum was then estimated for the marker enzyme, γ-GTP[19].

The rats were then killed by an overdose of anaesthetic ether and stomachs were cut along greater curvature. The gastric contents were then collected through the oesophagus and measured for volume. They were centrifuged at 3000 rpm for 20 min. The supernatant was subjected to analysis for titrable acidity and total volume of gastric juice[20]. The stomachs were opened along the greater curvature, and the mucosa was rinsed with cold normal saline to remove blood contaminant, if any. The sum of the length (mm) of all lesions for each stomach was used as the ulcer index (UI). In each rat, the macroscopic injury of each ulcer was scored by an independent observer according to a scale ranging from 0 to 4 as follows: (0) no macroscopic changes, (1) mucosal erythema only, (2) mild mucosal edema, slight bleeding or small erosions, (3) moderate edema, bleeding ulcers or erosions, and (4) severe ulceration, erosions, edema and tissue necrosis[21].

After examination, the stomachs were weighed and immediately immersed in alcian blue solution for determining the mucus wall thickness[22]. Only pylorus ligation was performed on one parallel group of animals which received only 1% CMC solution. The same parameters were checked in this group so as to differentiate the additional effects of aspirin upon pylorus ligated group. One parallel group received only 1% CMC in which pylorus ligation was not performed. All data are expressed as mean±standard error of the mean (SEM) of 8 rats per experimental group. The comparisons were done by ANOVA followed by Dunnett's t test.

Tables 1 and 2 depict that the aspirin+pylorus-ligated group showed significant increase in the serum γ-GTP levels, ulcer index and decrease in gastric wall mucus thickness as compared to normal control healthy rats (i.e. group I rats). Total acidity was increased but no significant change occurred in volume of gastric juice as compared to CMC+ pylorus ligated rats (i.e. group II rats). Administration of ginger oil in two doses of 0.5 g/kg, p.o. and 1 g/kg, p.o. resulted in significant reduction in serum γ-GTP levels, ulcer index, total acidity and increase in gastric wall mucus thickness. Less significant change was found in volume of gastric juice with the ginger oil dose of 0.5 g/kg; p.o. but there was no significant change in volume of gastric juice with the dose of 1 g/kg, p.o. as compared to aspirin+pylorus ligated group (group III rats). In the standard drug-treated rats (omeprazole 10 mg/kg /p.o. i.e. group VI rats), there was a significant decrease in serum γ-GTP levels, ulcer index, total acidity and increase in gastric wall mucus thickness but no significant change was found in volume of gastric juice as compared with aspirin+pylorus ligated rats (group III rats). Administration of ginger oil alone (group VII rats) resulted in significant decrease in serum γ-GTP level but no significant change was found in gastric wall mucus as compared with CMC+pylorus ligated rats (i.e. group II rats).

**TABLE 1:** EFFECT OF DIFFERENT DOSES OF GINGER OIL AND OMEPRAZOLE ON γ- GTP AND GASTRIC WALL MUCUS

Groups	Treatment (n=8)	γ-GTP (IU)	Gastric wall Mucus (μg/g of stomach wt.)
I	Vehicle control CMC [1 ml (1%)/kg/day, p.o]	20.8±1.0	181.36 ±18.50
II	CMC [1 ml (1%)/kg/day, p.o.] + pylorus ligation	86.76±2.86$$	151.38±14.72$$
III	Aspirin (200 mg/kg/day, p.o.) + pylorus ligation	95.98±11.90$$,∞	92.42±9.49$$,##
IV	Ginger Oil (0.5 g/kg/p.o./day) + aspirin (200 mg/kg/day, p.o.) + pylorus ligation	66.6±1.70**	193.56±12.39**
V	Ginger Oil (1 g/kg/day, p.o.) + aspirin (200 mg/kg/day, p.o.) + pylorus ligation	65.92 ±2.10**	152.72± 16.55**
VI	Omeprazole (10 mg/kg/day) + aspirin (200 mg/kg/day,p.o) + pylorus ligation	46.80±3.87**	226.31±15.64**
VII	Ginger oil per se (1 g/kg/day, p.o.)	49.78±3.43$$	155.95 ±13.29ns*

All values are expressed as mean±SEM;

* ns (non significant) as compared to vehicle control group (group I);

^ns∞^ as compared to CMC+pylorus ligated group (group II);

$$ *P*< 0.01, as compared to vehicle control group (i.e. group I);

** *P*<0.01, as compared to aspirin+pylorus ligation treated group (group III);

## *P*<0.01, as compared to CMC+pylorus ligated group (group II)

**TABLE 2:** EFFECT OF DIFFERENT DOSES OF GINGER OIL AND OMEPRAZOLE ON ULCER INDEX, TOTAL ACIDITY AND VOLUME GASTRIC JUICE

Groups	Treatment (n=8)	Ulcer Index Score	Total acidity (meq/lit)	Vol. of gastric Juice (ml/100g)
I	Vehicle control CMC [1 ml (1%)/kg/day, p.o]	0.00	-	-
II	CMC [1 ml (1%)/kg/day, p.o.] + pylorus ligation	10.64± 0.40$$	50.16±2.50	1.44 ±0.14
III	Aspirin (200 mg/kg/day, p.o.) + pylorus ligation	14.40± 0.40$$,##	80.00 ± 7.20##	1.37±0.04ns∞
IV	Ginger Oil (0.5 g/kg/p.o./day) + aspirin (200 mg/kg/day, p.o.) + pylorus ligation	11.31 ± 0.56**	42.50±3.90**	0.80± 0.083**
V	Ginger Oil (1 g/kg/day, p.o.) + aspirin (200 mg/kg/day, p.o.) + pylorus ligation	10.71 ± 0.52**	37.33±9.14**	1.40±0.19nsЄ
VI	Omeprazole(10 mg/kg/day) + aspirin (200 mg/kg/day,p.o) + pylorus ligation	6.50±0.52**	41.66±3.88**	1.10±0.04nsЄ
VII	Ginger oil per se (1 g/kg/day, p.o.)	0.00ns*	-	-

All values are expressed as mean±SEM;

*ns (non significant) as compared to vehicle control group (group I);

ns∞ as compared to CMC+pylorus ligated group (group II);

nsЄ as compared to aspirin +pylorus ligated group (group III);

$$ *P*<0.01, as compared to vehicle control group (group I);

## *P*<0.01, as compared to CMC+pylorus ligated group (group II);

** *P*<0.01,as compared to aspirin +pylorus ligated group (group III)

Aspirin+pylorus ligation-induced gastric ulcer model is a useful model to induce severe ulceration in experimental animals[23]. Aspirin causes mucosal damage by interfering with prostaglandin synthesis, increasing acid secretion and back diffusion of H^+^ ions[24]. The inhibition of mucosal prostaglandin production occurs rapidly following oral administration of aspirin. This is correlated with the rapid absorption of these drugs through the mucosa[16]. In pylorus ligation, the digestive effect of accumulated gastric juice and interference of gastric blood circulation are responsible for the induction of ulceration[25].

In the present study, oral treatment with test drug, viz. ginger oil in the two doses of 0.5 g/kg/p.o. and 1.0 g/kg/p.o. (group IV and group V rats, respectively) and the standard drug, i.e. omeprazole 10 mg/kg/p.o. (group VI rats) significantly (p<0.01) decreased the serum γ-GTP levels as compared to aspirin+pylorus ligated rats (group III rats). Decrease in γ-GTP levels were reported by Bhandari *et al*. (2003)[26] where they studied hepatoprotective activity of ethanol extracts of ginger on alcohol-induced hepatotoxicity in rats.

In the present study, treatment with ginger oil in the doses of 0.5 g/kg/p.o. and 1.0 g/kg/p.o. (group IV and group V rats, respectively) and the standard drug, omeprazole (10mg/kg/p.o.) i.e. group VI rats significantly (p<0.01) increased the mean gastric wall mucus thickness as compared to the aspirin+pylorus ligated rats (group III rats). Our study corroborates the findings of Dhuley (1999)[27] reported an increase in gastric wall mucus thickness (barrier mucus) in rats treated with *rhinax,* a herbal formulation consisting water extracts of *Withania somnifera* L. (root), *Asparagus racemosus* Willd. (root), *Mucuna prurience* (root), *Phyllanthus emblica* Gaertn. (fruit), *Myristica fragrance* Houtt. (seed), *Glycyrrhiza glabra* L. (root), against physical and chemical factors-induced gastric and duodenal ulcers in rats.

Increased mucus secretion by the gastric mucosal cells can prevent gastric ulceration by several mechanisms, including lessening of stomach wall friction during peristalsis and gastric contractions, improving the buffering of acid in gastric juice and by acting as an effective barrier to back diffusion of H^+^ ion[28].

A significant (p<0.01) decrease in mean ulcer index was seen in test drug treated rats (group IV and group V, respectively) and the standard drug treated rats (group VI rats) as compared to the aspirin+pylorus ligated rats (group III rats). The decrease in ulcer index is in conformity with those of Goel *et al,*[29] who reported the decrease in ulcer index on treatment with vegetable plantain banana in aspirin+pylorus ligated rats and Agarwal *et al,*[30] who also reported the decrease in ulcer index on treatment with *Piper longum* Linn, *Zingiber officianalis* Linn and *ferula* species in pylorus ligated rats. Many workers have established the relationship between acid production and peptic ulcers[31–33]. For several decades, the adage “no acid-no ulcer” and the drugs used to reduce acid secretion has dominated the pharmacological basis of ulcer therapy[34].

The aspirin+pylorus ligated rats, when treated with ginger oil at doses of 0.5 g/kg/p.o. and 1.0 g/kg/p.o. for a period of 5 days (30 min before aspirin treatment), it was reported that there has been significant (p<0.01) decrease in the mean total acidity in ginger oil treated rats (group IV and group V rats, respectively) as compared to untreated aspirin+pylorus ligated rats. When aspirin+pylorus ligated rats (group III rats) were treated with 10 mg/kg/p.o. of omeprazole (group VI rats), there was a decrease in mean total acidity and the decrease was significant (p<0.01). Our results corroborate the findings of Surender[35], who reported a decrease in total acidity on treatment with fixed oil of *Ocimum basilicum* Linn. in aspirin+pylorus ligated rats.

It has been studied that inhibition of gastric secretion protects the gastroduodenal mucosa against the injuries caused by pylorus ligatoin[36]. Sanmugapriya and Venkataraman[23] have shown that decrease in gastric juice volume reduce the total acidity and thus ulcer index. These studies support our results that the enhancement of production of gastric wall mucus, decrease in gastric juice volume reduces the total acidity and thus ulcer index elicited by ginger oil.

The results of the present study demonstrated that ginger oil treatment has antisecretory activity, as observed by the decrease in total acidity and volume of gastric juice. Further, the ginger oil treatment offers cytoprotection by increasing mucus wall thickness (barrier mucus) and by reducing the levels of serum γ-GTP. Therefore, it can be concluded that the ginger oil has a great potential to be used as a gastro protective drug in combination with other drugs or alone. A better understanding of the mechanisms and the active compounds present in this plant may, in the future, clarify the scientific bases of its use in folk medicine or even provide new alternatives for the clinical management of gastric ulcers.
